# Paediatric trauma on the Last Frontier: an 11-year review of injury mechanisms, high-risk injury patterns and outcomes in Alaskan children

**DOI:** 10.3402/ijch.v73.25066

**Published:** 2014-08-06

**Authors:** Christopher W. Snyder, Oliver J. Muensterer, Frank Sacco, Shawn D. Safford

**Affiliations:** 1 Department of Surgery, 6th Medical Group, MacDill Air Force Base, Tampa, FL, USA; 2 Department of Surgery, The Children's Hospital, Providence Alaska Medical Center, Anchorage, AK, USA; 3 Division of Acute Care Surgery, University of South Florida, Tampa, FL, USA; 4 Division of Paediatric Surgery, The Children's Hospital at Montefiore, Albert Einstein College of Medicine, Bronx, NY, USA; 5 Department of Surgery, Alaska Native Medical Center, Anchorage, AK, USA; 6 Division of Paediatric Surgery, Virginia Tech Carilion School of Medicine, Roanoke, VA, USA

**Keywords:** Alaska, injury, epidemiology, youth, resource utilization

## Abstract

**Background:**

Paediatric trauma system development in Alaska is complicated by a vast geographic coverage area, wide regional variations in environment and culture, and a lack of available published data.

**Objective:**

To provide a detailed description of paediatric trauma mechanisms, high-risk injury patterns and outcomes in Alaska.

**Design:**

This retrospective study included all children aged 17 years or younger in the State of Alaska Trauma Registry database admitted with traumatic injury between 2001 and 2011. Each injury record was reviewed individually and assigned a mechanism based on Centers for Disease Control E-codes. Geographic definitions were based on existing Emergency Medical Services regions. Mechanisms were compared by geographic region, patient demographics, injury characteristics and outcome. Subgroup analysis of fatal injuries was performed to identify causes of death.

**Results:**

Of 5,547 patients meeting inclusion criteria, the most common mechanisms of injury were falls (39%), motor vehicle collisions (10%) and all-terrain vehicle (ATV) accidents (9%). The overall case fatality rate was 2%. Mechanisms with the greatest risk of death were gunshot wounds (21%), pedestrians struck by motorized vehicles (9%) and motor vehicle collisions (5%). These 3 mechanisms accounted for 15% of injuries but 60% of deaths in the overall cohort. Injury patterns involving combined central nervous system (CNS) and torso injuries were unusual but especially lethal, occurring in 3% of patients but carrying a case fatality rate of 18%. Although the distribution of mechanisms was generally similar for each geographic region, ATV and snowmobile injuries were significantly more common in remote areas (23% remote vs. 7% non-remote, p < 0.0001).

**Conclusions:**

Mechanisms of paediatric trauma in Alaska have widely varying impacts on outcome and show some variation by region. Highest-risk mechanisms include gunshot wounds and motorized vehicle-related accidents. Prevention efforts should give special attention to CNS injury prevention, ATV and snowmobile safety in remote areas, and optimization of management of multisystem trauma. Further studies should investigate predictors of outcome in greater detail.

Trauma system development in Alaska is complicated by great distances, extremes of climate and terrain, and a large proportion of residents living in very rural and remote areas. Injuries remain a common cause of death for Alaskan children, especially those of Alaska Native ethnicity, for whom approximately 58% of deaths are injury related (1, 2). A team from the American College of Surgeons Committee on Trauma recently evaluated Alaska's trauma system, recommending more intensive study of paediatric trauma care needs with a goal of establishing a paediatric trauma centre of excellence (3). Since 1992, all injuries that are admitted to some level of medical care have been entered into a statewide trauma registry (4). However, little has been published regarding mechanisms and outcomes of trauma in Alaskan children. The purpose of this study was to describe specific mechanisms of injury in this population, with respect to their frequency, demographics, corresponding high-risk injury patterns and outcomes.

## Methods

All records for children aged 0–17 years who were injured between January 2001 and December 2011 were obtained from the Alaska Trauma Registry. Records were excluded if they were non-trauma mechanisms (hanging, foreign body aspiration/ingestion, poisoning, toxin exposure and isolated drowning), trivial injuries (Injury Severity Score [ISS] less than or equal to 1), isolated burn or cold injuries, or had an undefined injury location (n=2).

Registry variables of interest included patient age, sex, ethnicity, location and timing of injury occurrence, alcohol or drug use within 6 hours of injury, a brief narrative description of the injury, highest ISS, Center for Disease Control and Prevention (CDC) injury E-code, and up to 9 International Classification of Diseases, Ninth Revision, Clinical Modification (ICD-9) diagnosis codes. Geographic regions of injury occurrence were classified into 4 groups based on the Alaska Emergency Medical Services system as follows: Anchorage/Matanuska-Susitna (Mat-Su), Fairbanks (North Star Borough), Southeast Alaska and Remote (Aleutian-Pribilof, Bristol Bay, Copper River, Kenai, Kodiak, North Slope, Northwest Arctic, Norton Sound, Rural Interior and Yukon-Kuskokwim).

The precise mechanism of each injury was determined by the CDC E-code from the registry. The brief narrative description of each injury was compared to the assigned E-code to verify accuracy. Any discrepancies were resolved by modifying the E-code to match the narrative description. Intent (i.e. accidental vs. non-accidental) was often unknown or equivocally documented; consequently, no attempt was made to separate mechanisms by intent. Mechanisms were initially grouped as blunt or penetrating (gunshot wound, stab/cut/impalement). Blunt injuries were further categorized as motor vehicle-related or non-motor vehicle-related. Motor vehicle-related mechanisms consisted of motor vehicle occupant (driver or passenger in an on-road vehicle), motorcycle/dirt bike (driver or passenger on 2-wheeled vehicle), all-terrain vehicle (ATV) (driver or passenger on 3- or 4-wheeled off-road vehicle), snowmobile or snowmachine (driver or passenger), bicycle vs. motorized vehicle (struck by any motorized vehicle while riding a bicycle), and pedestrian vs. motorized vehicle (struck by any motorized vehicle while walking). Non-motor vehicle-related mechanisms were bicycle (rider or passenger), fall, sports-related (occurring in the context of a defined game or sport), struck by a person or non-motorized object, sledding, animal-related, and other (rare mechanisms not elsewhere classifiable; including water transport, explosion, airplane, strain/overexertion and nonspecific abuse/neglect). Due to the frequency and wide variety of falls, these were subcategorized by whether the fall was from a skateboard, a building, skis or snowboard, playground equipment or other.

Variables used to describe injury characteristics, resource utilization and outcome included ISS, presence of ISS of 15 or greater (severe injury), number of hospital days, total hospital charges, mortality and a composite of death or permanent disability (unfavourable outcome). Permanent disability is defined in the registry as the patient not being expected to ever return to a pre-injury level of function, based on the most recent hospital discharge notes.

High-risk injury patterns were defined based on ICD-9 codes. These patterns consisted of central nervous system (CNS) injury (800–804, 851–854, 870), injury to thoracic or abdominal organs (860–869), pelvic fracture (808) and vascular injury (900–904). Since these high-risk injury patterns typically require surgical evaluation, they have previously been described as “surgical diagnoses” (5). In order to maintain consistency of terminology with previous studies, extremity fractures and skin/soft tissue injuries were not considered to be high-risk diagnoses. Patients with high-risk injuries were grouped into 3 subcategories to better define the injury pattern: CNS, non-CNS (thoracoabdominal organ injury, pelvic fracture and/or vascular disruption) and combined CNS and non-CNS injuries.

Mechanisms of injury were compared by geographic region, patient age group, injury severity, probability of producing a high-risk injury pattern, resource utilization, and outcome. Subset analysis was performed on fatalities to evaluate the frequency of high-risk injury patterns, with a goal of elucidating causes of death. Univariate and bivariate statistics were calculated using Chi-square and Kruskal–Wallis tests for categorical and continuous variables, respectively. All statistical analyses were performed using SAS 9.2 (SAS Institute, Cary, NC).

## Results

A total of 5,547 records met inclusion criteria. Table I shows demographics and mechanisms of injury for the overall cohort and stratified by geographic area. The distribution of ethnicity varied significantly by geographic area. Children of Alaska Native/American Indian ethnicity constituted 19 and 20% of those injured in the Fairbanks and Anchorage areas, respectively, but 65% of those injured in Remote areas (p<0.0001). Recent alcohol use was significantly more prevalent in remote versus urban communities (6% vs. 3%, p=0.0005). Overall, the most common mechanisms of injury were non-specific falls (26%), motor vehicle occupants (10%) and ATVs (9%). Animal-related injuries were relatively rare, with only 69 occurrences (1% of all injuries) reported over the 11-year study period. Their frequency did not differ by geographic area. Non-motor vehicle-related mechanisms tended to be uniformly distributed across geographic areas. The only exceptions were falls from skis or snowboards and injuries resulting from being struck by a person or object, both of which were slightly less prevalent in remote communities (p<0.05).

**Table I:** Demographics and injury mechanisms, stratified by Emergency Medical Services geographic regions

	Overall (n=5,547)	Anchorage/Mat-Su (n=2,498)	Fairbanks (n=507)	Southeast (n=442)	Remote (n=2,100)
Age, years	12 (6–15)	12 (6–15)	12 (5–15)	12 (6–15)	12 (6–15)
Female sex	1,869 (34)	803 (32)	169 (33)	169 (38)	728 (35)
Ethnicity^a^					
Alaska Native/American Indian	2,148 (39)	504 (20)	97 (19)	175 (40)	1,372 (65)
Other	441 (8)	348 (14)	41 (8)	23 (5)	29 (1)
Unknown	24 (<1)	104 (4)	20 (4)	24 (5)	127 (6)
White	2,683 (48)	1,542 (62)	349 (69)	220 (50)	572 (27)
Recent alcohol use^a^	253 (5)	86 (3)	19 (4)	23 (5)	125 (6)
Recent drug use	184 (3)	84 (3)	11 (2)	21 (5)	68 (3)
Penetrating mechanisms					
Gunshot wound	116 (2)	54 (2)	7 (1)	3 (1)	52 (2)
Stab, cut, or impalement	66 (1)	29 (1)	11 (2)	5 (1)	24 (1)
Blunt mechanisms					
Motor vehicle-related					
Motor vehicle occupant^a^	529 (10)	277 (11)	58 (11)	59 (13)	135 (6)
Motorcycle/dirt bike^a^	183 (3)	77 (3)	26 (5)	9 (2)	71 (3)
All-terrain vehicle^a^	486 (9)	120 (5)	29 (6)	22 (5)	315 (15)
Snowmachine^a^	254 (5)	58 (2)	15 (3)	5 (1)	176 (8)
Bicycle vs. motorized vehicle	86 (2)	47 (2)	11 (2)	10 (2)	18 (1)
Pedestrian vs. motorized vehicle	201 (4)	90 (4)	7 (3)	18 (4)	86 (4)
Non-motor vehicle-related					
Bicycle	374 (7)	192 (8)	32 (6)	26 (6)	124 (6)
Fall – from skateboard	92 (2)	57 (2)	4 (1)	6 (1)	25 (1)
Fall – from building	139 (3)	66 (3)	12 (2)	11 (2)	50 (2)
Fall – from skis/snowboard^a^	197 (4)	131 (5)	23 (5)	19 (4)	24 (1)
Fall – from playground equipment	250 (5)	110 (4)	18 (4)	20 (5)	102 (5)
Fall – other	1,464 (26)	645 (26)	142 (28)	138 (31)	539 (26)
Sports	414 (7)	200 (8)	48 (9)	34 (8)	132 (6)
Struck by person or object (non-motorized)^a^	358 (6)	188 (8)	31 (9)	27 (6)	112 (5)
Sledding	108 (2)	60 (2)	14 (3)	12 (3)	22 (1)
Animal-related	69 (1)	34 (1)	4 (1)	3 (1)	28 (1)
Other^b^	161 (3)	63 (3)	18 (4)	15 (3)	65 (3)

Data presented as N (%) and median (interquartile range) for categorical and continuous variables, respectively.

a Statistically significant difference between regions (p<0.05) on Chi-square and Kruskal–Wallis tests for categorical and continuous variables, respectively.

b Other category includes water transport, explosion, airplane crash, strain/overexertion and abuse/neglect not elsewhere classifiable.

Motor vehicle-related mechanisms showed geographic variations. In urban regions, ATV and snowmobile injuries constituted 7–9% of the cohort, while in small rural regions they made up 23% (p<0.0001). Conversely, motor vehicle occupants made up 11–13% of the cohort in urbanized areas but only 6% in small rural regions (p<0.0001).

Mechanisms of injury varied significantly by age group, as demonstrated in Table II. Non-specific falls were a common mechanism in all age groups, but their frequency declined with increasing age, from 52% in very young children (age 0–4) to 11% among older adolescents (age 15–17). In school-age children (age 5–9), falls from playground equipment were common, accounting for 13% of injuries. Sports (11%) and bicycle (10%) injuries were more prevalent among young adolescents (age 10–14). Mechanisms involving operating or riding motorized vehicles predominated among older adolescents, accounting for 45% of injuries. Sports-related injuries (12%) were the most common non-motor vehicle-related mechanism in the older adolescent age group. Although penetrating mechanisms were relatively rare across all age groups, gunshot wounds accounted for 66 injuries (4%) among older adolescents.

**Table II:** Mechanisms of injury, by age group^a^

	0–4 years (n=1,074)	5–9 years (n=1,113)	10–14 years (n=1,726)	15–17 years (n=1,628)
Penetrating mechanisms				
Gunshot wound	4 (<1)	8 (1)	37 (2)	66 (4)
Stab, cut, or impalement	12 (1)	10 (1)	14 (1)	30 (2)
Blunt mechanisms				
Motor vehicle-related				
Motor vehicle occupant	50 (9)	59 (5)	91 (5)	328 (20)
Motorcycle/dirt bike	0	8 (1)	93 (5)	82 (5)
All-terrain vehicle	19 (2)	58 (5)	208 (12)	201 (12)
Snowmachine	8 (1)	21 (2)	88 (5)	136 (8)
Bicycle vs. motorized vehicle	4 (<1)	20 (2)	43 (2)	19 (1)
Pedestrian vs. motorized vehicle	55 (5)	57 (5)	46 (3)	42 (3)
Non-motor vehicle-related				
Bicycle	13 (1)	58 (5)	176 (10)	65 (4)
Fall – from skateboard	2 (<1)	7 (1)	47 (3)	36 (2)
Fall – from building	70 (7)	34 (3)	24 (1)	11 (1)
Fall – from skis/snowboard	1 (<1)	18 (2)	97 (6)	81 (5)
Fall – from playground equipment	46 (4)	141 (13)	57 (3)	6 (<1)
Fall – other	559 (52)	393 (35)	339 (20)	172 (11)
Sports	7 (1)	22 (2)	190 (11)	195 (12)
Struck by person or object (non-motorized)	147 (14)	53 (5)	68 (4)	90 (6)
Sledding	7 (1)	39 (4)	42 (2)	20 (1)
Animal-related	13 (1)	23 (2)	25 (1)	8 (<1)
Other^b^	57 (5)	22 (2)	41 (2)	40 (2)

Data presented as N (%).

a All mechanisms demonstrated statistically significant variation by age group (p<0.05) on Chi-square test.

b Other category includes water transport, explosion, airplane, strain/overexertion and abuse/neglect not elsewhere classifiable.

Table III compares mechanisms by injury severity, resource utilization, and outcome. In general, penetrating and motor vehicle-related mechanisms carried greater impact than non-motor vehicle-related mechanisms. Gunshot wounds tended to be high severity injuries (ISS≥15 in 30%), required the longest hospital stay (median 4 days) and hospital charges (median $24,100), and carried the highest risk of fatality (21%) or unfavourable outcome (26%). In contrast, falls from playground equipment were low severity events (ISS≥15 in 2%) and carried minimal risk of death (0%) or unfavourable outcome (1%). The mechanisms tending to result in higher severity and greater risk of poor outcome among non-motor vehicle-related mechanisms were falls from buildings, animal-related injuries and being struck by a person or non-motorized object.

**Table III:** Comparison of mechanisms by injury severity, resource utilization and outcome^a^

	Injury Severity Score (ISS)	ISS≥15	Hospital days	Hospital charges, thousands of $US	Death	Unfavourable outcome
Penetrating						
Gunshot wound	9 (4–16)	34 (30)	4 (1–8)	24.1 (10.2–52.9)	24 (21)	30 (26)
Stab, cut, or impalement	4 (4–9)	4 (6)	2 (1–5)	12.7 (6.5–29.5)	0	0
Blunt						
Motor vehicle-related						
Motor vehicle occupant	9 (5–17)	153 (30)	3 (1–6)	16.9 (8.1–38.8)	28 (5)	73 (14)
Motorcycle/dirt bike	5 (4–9)	25 (14)	2 (1–5)	14.2 (7.3–30.7)	2 (1)	13 (7)
All–terrain vehicle	5 (4–9)	58 (12)	2 (1–4)	11.3 (4.8–23.3)	10 (2)	20 (4)
Snowmachine	5 (4–9)	21 (8)	2 (1–4)	9.8 (4.2–25.4)	1 (<1)	5 (2)
Bicycle vs. motorized vehicle	9 (8.5–14)	15 (18)	3 (1–6)	17.4 (7.8–38.8)	3 (3)	11 (13)
Pedestrian vs. motorized vehicle	9 (4–13)	39 (20)	2 (1–4)	12.0 (4.8–26.8)	18 (9)	23 (11)
Non-motor vehicle-related						
Bicycle	4 (4–9)	27 (8)	1 (1–3)	9.0 (4.6–19.1)	3 (1)	6 (2)
Fall – from skateboard	4 (4–9)	5 (5)	1 (1–2)	9.5 (6.4–14.3)	0	0
Fall – from building	5 (4–10)	22 (16)	1 (1–3)	10.3 (4.9–21.1)	1 (1)	6 (4)
Fall – from skis/snowboard	4 (4–9)	13 (7)	1 (1–3)	9.9 (6.3–21.2)	0	3 (2)
Fall – from playground equipment	4 (4–5)	4 (2)	1 (1–2)	7.0 (4.1–12.4)	0	2 (1)
Fall – other	4 (4–9)	79 (6)	1 (1–2)	7.5 (4.1–13.5)	5 (<1)	25 (2)
Sports	4 (4–4)	18 (5)	1 (1–2)	10.4 (5.5–18.8)	1 (<1)	3 (1)
Struck by person or object (non–motorized)	4 (4–9)	49 (15)	2 (1–3)	8.4 (4.3–17.8)	10 (3)	21 (6)
Sledding	8 (4–9)	7 (7)	2 (1–4)	13.0 (5.6–27.6)	2 (2)	7 (6)
Animal–related	9 (4–10)	9 (13)	2 (1–6)	16.7 (6.9–43.0)	2 (3)	5 (7)
Other^b^	4 (4–9)	12 (8)	1 (1–3)	6.8 (3.4–19.6)	6 (4)	14 (9)

Data presented as N (%) and median (interquartile range) for categorical and continuous variables, respectively.

a All variables demonstrated statistically significant variation by mechanism (p<0.05) on Chi-square and Kruskal–Wallis tests for categorical and continuous variables, respectively.

b Other category includes water transport, explosion, airplane, strain/overexertion and abuse/neglect not elsewhere classifiable.

Frequencies of high-risk injury patterns, for the entire cohort and for each mechanism individually, are provided in Table IV. The most common high-risk pattern was CNS injury (21% of all injuries), followed by abdominal organ injury (8%). Mechanisms most likely to produce a CNS injury included being struck by a person or non-motorized object (39%), motor vehicle occupant (38%) and bicycle vs. motorized vehicle (28%). Thoracic and abdominal organ injuries were most prevalent among motor vehicle occupants, stab wounds and gunshot wounds. Pelvic fractures were uncommon, but had their highest prevalence among pedestrians struck by motorized vehicles (10%). Vascular injuries were generally rare among blunt mechanisms. The only exception was animal-related injuries, for which the prevalence was 6%, comparable to the 9 and 6% prevalence among stab wounds and gunshot wounds, respectively. Combined CNS and non-CNS injury patterns occurred most frequently in motor vehicle occupants (13%), bicycle vs. motorized vehicle (7%) and pedestrian vs. motorized vehicle (7%).

**Table IV:** Comparison of mechanisms by prevalence of high-risk injury patterns^a^

		Non-CNS torso injury				
	CNS injury	Thoracic organ	Abdominal/ pelvic organ	Pelvic fracture	Vascular disruption	Combined CNS and non-CNS injury
Overall	1,179 (21)	323 (6)	435 (8)	133 (2)	39 (1)	142 (3)
Penetrating						
Gunshot wound	27 (23)	22 (19)	17 (15)	0	7 (6)	2 (2)
Stab, cut, or impalement	5 (8)	14 (21)	11 (17)	0	6 (9)	1 (2)
Blunt						
Motor vehicle-related						
Motor vehicle occupant	202 (38)	112 (21)	114 (22)	49 (9)	5 (1)	69 (13)
Motorcycle/dirt bike	24 (13)	14 (8)	25 (14)	4 (2)	3 (2)	5 (3)
All-terrain vehicle	124 (26)	36 (7)	39 (8)	20 (4)	4 (1)	10 (2)
Snowmobile	44 (17)	15 (6)	14 (6)	10 (4)	2 (1)	4 (2)
Bicycle vs. motorized vehicle	24 (28)	15 (17)	7 (8)	8 (9)	0	6 (7)
Pedestrian vs. motorized vehicle	48 (24)	29 (14)	25 (12)	20 (10)	0	14 (7)
Non-motor vehicle-related						
Bicycle	67 (18)	4 (1)	49 (13)	0	1 (<1)	4 (1)
Fall – from skateboard	14 (15)	0	3 (3)	0	0	0
Fall – from building	48 (35)	9 (6)	6 (4)	2 (1)	0	7 (5)
Fall – from skis/snowboard	10 (5)	5 (3)	26 (13)	7 (4)	1 (1)	2 (1)
Fall – from playground equipment	16 (6)	0	5 (2)	0	3 (1)	0
Fall – other	287 (20)	12 (1)	25 (2)	3 (<1)	2 (<1)	3 (<1)
Sports	36 (9)	8 (2)	26 (6)	4 (1)	0	0
Struck by person or object (non-motorized)	139 (39)	9 (3)	24 (7)	3 (1)	0	6 (2)
Sledding	21 (19)	5 (5)	7 (6)	0	0	2 (2)
Animal-related	17 (25)	6 (9)	6 (9)	0	4 (6)	2 (3)
Other^b^	26 (16)	8 (5)	6 (4)	3 (2)	1 (1)	5 (3)

Data presented as N (%). CNS, central nervous system.

a All injury patterns demonstrated statistically significant variation by mechanism (p<0.05) on Chi-square test.

b Other category includes water transport, explosion, airplane crash, strain/overexertion and abuse/neglect not elsewhere classifiable.

Table V shows the prevalence of high-risk injury patterns among children who died of their injuries. The most common pattern among fatalities was CNS injury (73%), followed by thoracic organ injury (28%). Combined CNS and non-CNS injuries were present in 22% of fatalities.

**Table V:** Prevalence of high-risk injury patterns among fatalities

High-risk injury pattern	Frequency among fatalities (n=116)
CNS injury	85 (73)
Non-CNS torso injury	
Thoracic organ	32 (28)
Abdominal/pelvic organ	20 (17)
Pelvic fracture	7 (6)
Vascular disruption	5 (4)
Combined CNS + non-CNS injury	26 (22)

Data presented as N (%). CNS, central nervous system.

## Discussion

This study provides an extensive overview of the largest cohort of paediatric trauma patients in Alaska to date. Mechanisms of injury varied widely across different demographics. As expected in such a large and geographically diverse state as Alaska, injury mechanisms reflect the characteristics of their respective geographic areas. More off-road than on-road motorized vehicle injuries occurred in remote areas, a finding consistent with previous studies (6). Age group distributions are similar to the rest of the United States (7). The high prevalence of CNS injury among paediatric trauma victims as a whole, and especially among fatalities, highlight the high impact of paediatric CNS injury demonstrated in other populations (8).

This study's findings are expected to be useful in both public health and clinical medical practice. Armed with the knowledge of which mechanisms are most common and which have the greatest impact on resource utilization and outcome, Alaskan public health officials can better focus their resources on primary and secondary prevention. The frequency and morbidity of motor vehicle-related injuries supports the on-going need for safety education programs and vehicle-appropriate protective equipment use such as seatbelts, car seats and helmets. Such programs should reflect the makeup of the community, placing greater emphasis on off-road vehicle use in rural areas. Though uncommon, the high morbidity and mortality associated with gunshot wounds in the paediatric population underscores the need for on-going prevention efforts for firearm injuries (9). The “struck by person or non-motorized object” mechanism likely includes a large proportion of non-accidental trauma, accounting for the very high proportion of CNS injuries (39%) produced by this mechanism. Previous studies have demonstrated that morbidity of non-accidental trauma exceeds that of accidental trauma (10, 11). Although not specifically addressed by this study, interpersonal violence and substance abuse are endemic problems in Alaska that require on-going attention and further study (12, 13). The combined CNS and non-CNS injury pattern occurred in only 3% of the overall cohort but was present in 22% of fatalities, reinforcing the danger this pattern poses and the need for effective prevention and treatment strategies.

Clinical practitioners who care for paediatric trauma patients may find it valuable to understand which injury patterns a given mechanism is likely to produce. Knowledge of pre-test probabilities for specific injuries may facilitate more judicious use of imaging modalities in the trauma workup for a given mechanism. More focused use of radiographic imaging could also help decrease problematic ionizing radiation exposure in children (14), while better correlation of mechanism of injury with the prevalence of high-risk injury patterns may facilitate triage and transfer decisions. This is especially relevant for head trauma in remote areas where limited diagnostic modalities are available and emergent air transport itself carries relatively high risk (15). The need for evidence-based trauma guidelines appropriate to Alaska's unique environment has long been recognized by the state trauma committee. In 2003, the committee convened a Head Trauma Guideline Task Force to develop clinical guidelines for computed tomography scanning, neurotrauma consultation, and transfer for head-injured adults and children (unpublished document).

The findings of this study are subject to some limitations. Registry data may suffer from incomplete or equivocal documentation, resulting in information bias. To minimize information bias, each record was manually verified against its narrative description to ensure accurate and precise E-code assignment. Since the distinction between accidental and non-accidental injury was often equivocally documented, categorization was done by objective mechanism alone, with no attempt made to distinguish mechanisms by intent. The quality of state registry data could potentially be improved by more rigorous categorization of injury intent in the future. Survival bias may also influence the results. Since deaths in the field are not routinely entered into the registry, some very severe injuries may have been excluded from analysis.

Despite its limitations, this large series serves as an important reference and a starting point for future studies in this population. More detailed analyses of motorized vehicle injuries are needed to delineate patterns of protective equipment use, such as seatbelts and helmets. High-risk injury patterns should be more closely evaluated to define better predictors of occurrence and possible mitigating factors, such as the accessibility of timely and appropriate trauma care. Such data will provide valuable guidance as Alaska's paediatric trauma system continues to be developed and refined.
